# The mitochondrial genome sequences of the round goby and the sand goby reveal patterns of recent evolution in gobiid fish

**DOI:** 10.1186/s12864-017-3550-8

**Published:** 2017-02-16

**Authors:** Irene Adrian-Kalchhauser, Ola Svensson, Verena E. Kutschera, Magnus Alm Rosenblad, Martin Pippel, Sylke Winkler, Siegfried Schloissnig, Anders Blomberg, Patricia Burkhardt-Holm

**Affiliations:** 1 Program Man-Society-Environment, Department of Environmental Sciences, University of Basel, Vesalgasse 1, Basel, 4051 Switzerland; 2 Department of Biological and Environmental Sciences, University of Gothenburg, Medicinaregatan 18A, 41390 Göteborg, Sweden; 3 The Linnaeus Centre for Marine Evolutionary Biology, University of Gothenburg, P.O. Box 460, 40530 Gothenburg, Sweden; 4 Department of Evolutionary Biology, Evolutionary Biology Centre, Uppsala University, Norbyvägen 18D, 75236 Uppsala, Sweden; 5 Department of Marine Sciences, NBIS Bioinformatics Infrastructure for Life Sciences, University of Gothenburg, Medicinaregatan 9C, 41390 Gothenburg, Sweden; 6 Heidelberg Institute for Theoretical Studies, Schloss-Wolfsbrunnenweg 35, 69118 Heidelberg, Germany; 7 Max Planck Institute of Molecular Cell Biology and Genetics, Pfotenhauerstrasse 108, 01307 Dresden, Germany; 8 Department of Marine Sciences, University of Gothenburg, Medicinaregatan 9C, 41390 Gothenburg, Sweden; 9 Department of Biological Sciences, University of Alberta, 11455 Saskatchewan Drive, Edmonton, AB Canada

**Keywords:** Mitogenome, Genome size, Genome organisation, Phylogeny, Gobiidae, Salinity, *Neogobius melanostomus*, *Pomatoschistus minutus*, *Ponticola kessleri*

## Abstract

**Background:**

Vertebrate mitochondrial genomes are optimized for fast replication and low cost of RNA expression. Accordingly, they are devoid of introns, are transcribed as polycistrons and contain very little intergenic sequences. Usually, vertebrate mitochondrial genomes measure between 16.5 and 17 kilobases (kb).

**Results:**

During genome sequencing projects for two novel vertebrate models, the invasive round goby and the sand goby, we found that the sand goby genome is exceptionally small (16.4 kb), while the mitochondrial genome of the round goby is much larger than expected for a vertebrate. It is 19 kb in size and is thus one of the largest fish and even vertebrate mitochondrial genomes known to date. The expansion is attributable to a sequence insertion downstream of the putative transcriptional start site. This insertion carries traces of repeats from the control region, but is mostly novel. To get more information about this phenomenon, we gathered all available mitochondrial genomes of Gobiidae and of nine gobioid species, performed phylogenetic analyses, analysed gene arrangements, and compared gobiid mitochondrial genome sizes, ecological information and other species characteristics with respect to the mitochondrial phylogeny. This allowed us amongst others to identify a unique arrangement of tRNAs among Ponto-Caspian gobies.

**Conclusions:**

Our results indicate that the round goby mitochondrial genome may contain novel features. Since mitochondrial genome organisation is tightly linked to energy metabolism, these features may be linked to its invasion success. Also, the unique tRNA arrangement among Ponto-Caspian gobies may be helpful in studying the evolution of this highly adaptive and invasive species group. Finally, we find that the phylogeny of gobiids can be further refined by the use of longer stretches of linked DNA sequence.

**Electronic supplementary material:**

The online version of this article (doi:10.1186/s12864-017-3550-8) contains supplementary material, which is available to authorized users.

## Background

Gobiids (Teleostei, Gobiidae, *sensu* Gill and Mooi 2011 [1]) are a diverse and fascinating group of small, predominately bottom-dwelling fish species with a world-wide distribution. The Gobiidae family contains more than 1700 species in more than 200 genera and is therefore one of the largest vertebrate families [2, 3]. Gobiids display a wide range of very special adaptations. Several species are able to breathe air and display an amphibious lifestyle. Other species spend early and late life stages at different salinities, or are euryhaline and can cope with sudden salinity shifts [2]. Some species display alternative reproductive tactics [4, 5]. Others are tremendously successful bioinvaders [6]. In recent years, two Eurasian gobiid species received particular attention. The sand goby *Pomatoschistus minutus* became an evolutionary model species for behavioural studies [4, 7–11], while the invasive round goby *Neogobius melanostomus* became a model species for invasion ecology and invasion genetics [6, 12–14].

Most West Eurasian gobiid species, including the sand goby and the round goby, lack molecular resources. Therefore, West Eurasian gobiids are under-represented in some phylogenetic studies. Sequence-based phylogenies based on up to five nuclear and mitochondrial markers have outlined two major clades within Gobiidae, the gobiine-like and the gobionelline-like gobiids (*sensu* Agorreta et al. [15]) [3, 16, 17]. West Eurasian gobiid species cluster with both clades. While the round goby and its Ponto-Caspian relatives belong to the gobiine-like gobiids [16], the sand goby group [18] belongs to the gobionelline-like gobiids [17].

Complete mitochondrial genomes can provide both sequence- and non-sequence based phylogenetic information of high resolution. A high rate of sequence evolution, a lack of recombination, its inheritance as one locus, and a short coalescence time compared to bi-parentally inherited nuclear loci [19] render the mitochondrial genome a valuable sequence-based phylogenetic marker. Also, size variations and non-coding sequence insertions and deletions [20, 21] as well as gene order variations between species and taxonomic groups [22, 23] are common and provide additional non-sequence based phylogenetic information. To confirm and to better understand phylogenetic relationships, molecular trees can then be interpreted with regard to physiological and ecological traits to identify branch-specific and ancestral adaptations [24].

The aim of this paper is to enhance our molecular understanding of the round and the sand goby by reporting the annotated mitochondrial genomes of these two West Eurasian gobiid research model species, and to place these genomes within the mitochondrial phylogeny of Gobiidae using forty Gobiidae species, nine species from other gobioid families, and of *Siganus guttatus* and *Lactoria diphana*. We extend current phylogenies using the additional power of gene arrangement analyses and whole mitochondrial genome tree building, and make sense of these phylogenies by linking them to the ecological properties of the analysed species. We also aim to identify novel molecular markers and to explore the origin and the potential implications of a novel, large non-coding sequence insertion in the round goby mitochondrial genome.

In this paper, we use the term “non-coding region” for all sequence of unknown function which is not coding for either protein or tRNAs. We use the term “control region” to refer to non-coding sequences which contain functional elements that control mitochondrial genome replication.

## Results

### Mitochondrial genome size

**Fig. 1:**
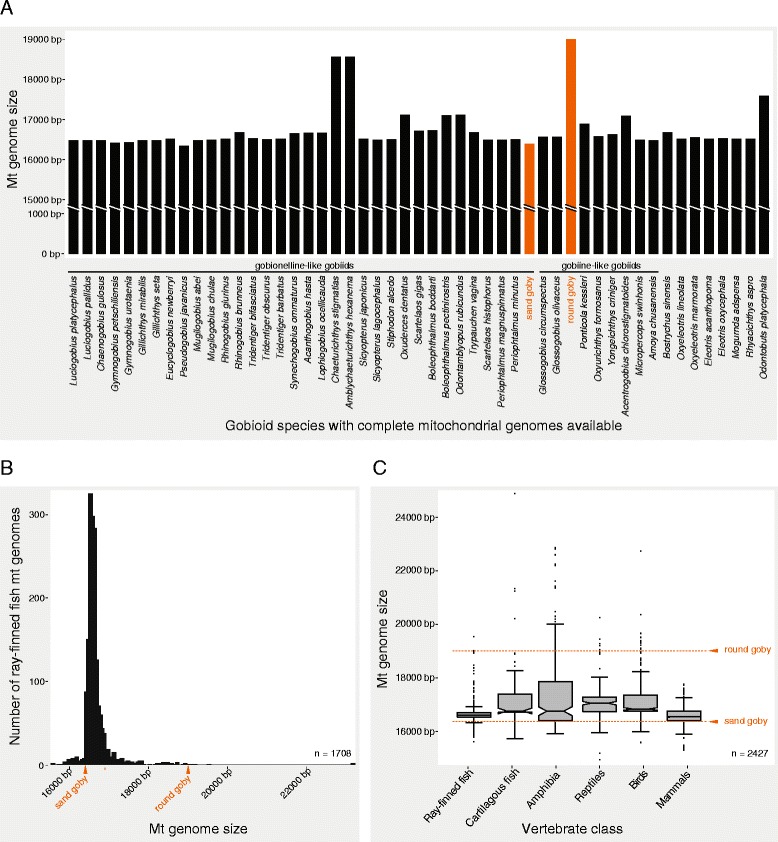
The round goby mitochondrial genome is one of the largest vertebrate mitochondrial genomes. **a** Barplot of gobioid mitochondrial genome sizes available on NCBI. Species are ordered according to phylogenetic relatedness as determined in this study. Round goby and sand goby genomes are highlighted in orange. **b** Histogram of mitochondrial genome sizes of 1708 ray-finned fish for which complete mitochondrial genomes were available. *Orange arrowheads* indicate the sizes of sand goby and round goby mitochondrial genomes. **c** Boxplots illustrating the mitochondrial genome size distributions for major vertebrate classes. For 2427 species, mitochondrial genomes as well as class annotations were available from NCBI and IUCN respectively. “Reptiles” includes all members of the reptilia clade (turtles, lizards, snakes, crocodiles etc.) except birds. *Orange arrowheads* and *dotted lines* indicate the sizes of sand goby and round goby mitochondrial genomes

**Fig. 2:**
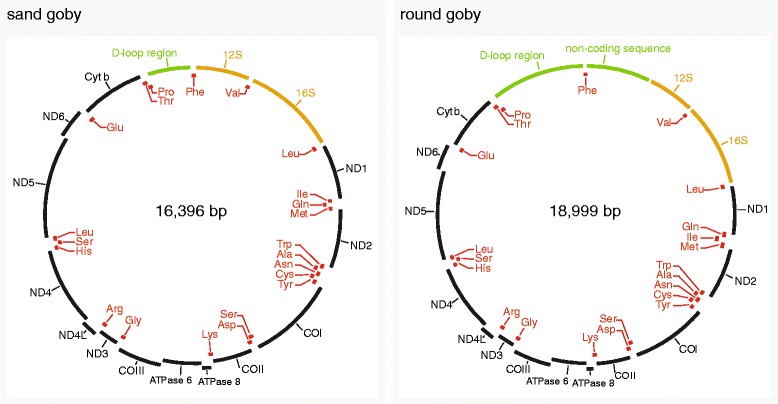
Sand and round goby mitochondrial genome gene arrangements. *Left* panel, sand goby mitochondrial genome annotation. *Right* panel, round goby mitochondrial genome annotation. The round goby genome features an enlarged D-loop region, a non-coding sequence insertion downstream of tRNA Phe, and an inverted arrangement of tRNA Ile and tRNA Gln

During genome sequencing projects for the round goby and the sand goby, we found that both the sand goby and the round goby mitochondrial genome were unusual with regard to their lengths. The sand goby mitochondrial genome (16,396 bp) was the second smallest mitochondrial genome of all gobiid species sequenced to date, while the round goby mitochondrial genome (18,999 bp) was the longest of all gobiid mitochondrial genomes known (Fig. 1a). The round goby mitochondrial genome also was larger than most mitochondrial genomes of ray-finned fish (Fig. 1b; Additional file 1: Table S2) and other vertebrates (Fig. 1c; Additional file 1: Table S3). Only 1.83% of sequenced vertebrate species (*n*_(total)_ = 3652) and 3.18% of sequenced animal species (*n*_(total)_ = 5567) had larger mitochondrial genomes. We found that the large size was caused by two instances of sequence insertions. First, the noncoding region between tRNA Proline and tRNA Phenylalanine that contains the control region was larger than in other species (1920 bp in the round goby, compared to 720 bp in sand goby). Second, the round goby genome featured a 1250 bp insert between tRNA Phenylalanine and the 12S rRNA gene (Fig. 2).

### Characterisation of non-coding sequence inserts

To better understand the source of the inserted sequences, we annotated functional regions and repeats. We identified the control region, which directs mitochondrial genome replication and transcription, by searching for Transcription Associated Site (TAS)- and Conserved Sequence Block (CSB)-like elements in conserved regions between tRNA Proline and tRNA Phenylalanine. Three canonical TAS sites are conserved in gobiids (Additional file 2: Figure S1 and Additional file 3: Figure S3). In addition, the round goby mitochondrial genome contains many canonical TAS sites in the context of tandem repeats. CSB sites were more difficult to identify. Only when relaxing search criteria (larger spacer sequence or relieved identity constraints on the third base), we were able to identify CSB-I like sequences in conserved regions downstream from the TAS motif. We also identified CSB II and CSB III-like sequences in conserved regions (Additional file 2: Figure S1).

**Fig. 3:**
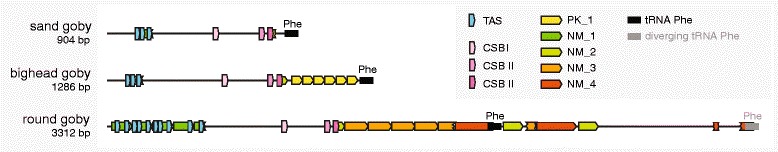
Architecture of non-coding sequences. Non-coding regions between tRNA Pro and the 12S gene of sand goby, bighead goby (the closest sequenced relative of round goby), and round goby are drawn to scale below each other. Conserved and/or putatively functional motifs of the control region such as TAS and CSB elements, tandem repeats, repeated sequences, and the location of tRNA Phe are indicated by *coloured bars*

The round goby non-coding sequence insert between tRNA Phenylalanine and the 12S rRNA gene did not contain TAS or CSB motifs, and was not similar to any published sequence, but bore similarity to sequence parts upstream of tRNA Phenylalanine. By mapping repeated sequence motifs between sand goby, round goby, and its closest sequenced relative, the bighead goby *Ponticola kessleri*, we identified five repeated motifs, which we called NM_1 (106 bp), NM_2 (100 bp), NM_3 (120 bp), NM_4 (202 bp), and PK_1 (56 bp; Fig. 3; Additional file 4: Figure S2). NM_3 and PK_1 are both located between CSB III and tRNA Phenylalanine and share a 14 bp core motif (TAATAATCATTTTA in bighead goby, TAATAATACATTTTTA in round goby). NM_4, NM_3, and NM_2 occur both upstream and downstream of tRNA Phenylalanine. All repeat motifs are predicted to form secondary structures (Additional file 4: Figure S2).

### Gene arrangement analysis

To compare gene arrangements between the analysed species, we performed automatic annotations. They revealed two instances of non-canonical gene arrangements involving tRNA genes, one in Ponto-Caspian gobies and one in *Odontobutis platycephala* (Additional file 3: Figure S3). In most gobioids analysed in this study, the tRNAs for Isoleucine, Glutamine and Methionine come in the order Ile/Gln/Met. In the two Ponto-Caspian species, the round goby and the bighead goby, these tRNAs are arranged as Gln/Ile/Met (Fig. 2). Strandedness differed between the two species, with Gln (-)/Ile (+)/Met (+) in the bighead goby and Gln (+)/Ile (-)/Met (+) in the round goby. In *Odontobutis platycephala*, we found that the tRNAs for Serine, Leucine and Histidine came as Ser/Leu/His instead of the canonical His/Ser/Leu arrangement (see also [25]).

### Mitochondrial phylogeny

**Fig. 4:**
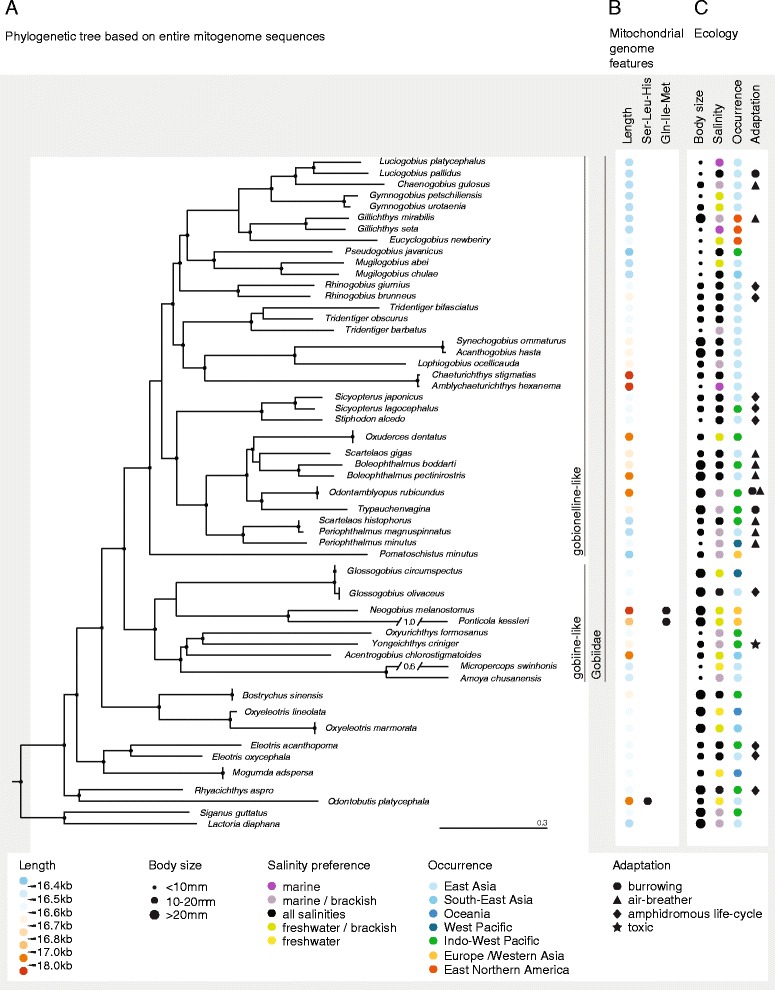
Phylogenetic analysis matched with biological and ecological species characteristics. **a** Phylogenetic tree of twelve concatenated mitochondrial genes obtained from MrBayes. *Black dots* indicate branches with a posterior probability value from MrBayes > 0.95 and a bootstrap value from maximum likelihood analysis in RAxML >75. **b** Mitochondrial genome features of the respective species. **c** Ecological characteristics of the respective species. Features and characteristics in **b** and **c** are indicated by the colour and/or size of the dot and/or symbol. Body size corresponds to maximal total length (TL) observed

To complement previous gene-centered studies, we buildt whole mitochondrial genome phylogenies using Bayesian and Maximum Likelihood approaches. The resulting phylogenies were consistent with phylogenies from previous studies (Fig. 4), with two exceptions (the placement of *Micropercops swinhonis* and *Oxyurichthys formosanus*, see below). As expected, we identified two major clades within Gobiidae, gobionelline-like gobiids and gobiine-like gobiids (Fig. 4; *sensu* Agorreta et al. [15]). Members from Butidae, Eleotridae, Rhyacichththydae, and Odontobutidae clustered as sister groups to the Gobiidae (Table 1).

**Table 1:** Comparison of this and previous efforts to build a Gobiidae phylogeny

Species name	This study	Thacker 2015 [37] (nuc + mtDNA genes)	Thacker 2014 [74] (mtDNA genes)	Tornabene et al. 2013 [3] (nuc genes, concatenated)	Thacker 2013 [17] (mtDNA genes, combining Thacker 2009 [73] and Thacker and Roje 2011) [40]	Agorreta et al. 2013 [15] (nuc + mtDNA genes)	Neilson et al. 2009 [16] (COI)
*Boleophthalmus pectinirostris*	gobionelline-like, Periophthalmus lineage	–	–	–	–	gobionelline-like, Periophthalmus lineage	–
*Bostrychus sinensis*	outgroup, Butidae	–	Butidae	–	–	–	–
*Eucyclogobius newberryi*	gobionelline-like, Acanthogobius lineage	gobionelline-like, Acanthogobius group	gobionelline-like	–	gobionelline-like, Northern Pacific group	gobionelline-like, Acanthogobius lineage	Gobionellinae, Sicydiinae
*Gillichthys mirabilis*	gobionelline-like, Acanthogobius lineage	gobionelline-like, Acanthogobius group	gobionelline-like	–	gobionelline-like, Northern Pacific group	gobionelline-like, Acanthogobius lineage	Gobionellinae, Sicydiinae
*Gymnogobius urotaenia*	gobionelline-like, Acanthogobius lineage	–	–	–	–	gobionelline-like, Acanthogobius lineage	–
*Mogurnda adspersa*	outgroup, Eleotridae	–	Eleotridae	–	–	Eleotrididae (*Mogurnda*sp.)	Xenisthmidae, Eleotridae
*Mugilogobius chulae*	gobionelline-like, Mugilogobius lineage	–	gobionelline-like	gobionelline-like, Mugilogobius group	–	–	–
*Neogobius melanostomus*	gobiine-like, Benthophilinae	gobiine-like, Gobius group	–	gobiine-like, Benthophilini	–	–	Benthophilinae, Neogobiini
*Odontamblyopus rubicundus*	gobionelline-like, Periophthalmus lineage	–	–	–	–	gobionelline-like, Periophthalmus lineage (Odontamblyopus sp.)	Oxudercinae, Amblyopinae
*Odontobutis platycephala*	outgroup, Odontobutidae	–	–	–	–	–	Odontobutidae
*Oxuderces dentatus*	gobionelline-like, Periophthalmus lineage	–	–	–	–	gobionelline-like, Periophthalmus lineage	–
*Oxyeleotris lineolata*	outgroup, Butidae	Butidae, outgroup	–	–	outgroup	–	–
*Oxyeleotris marmorata*	outgroup, Butidae	–	Butidae	–	–	Butidae	–
*Pomatoschistus minutus*	gobionelline-like, Pomatoschistus	gobionelline-like, Pomatoschistus group	–	–	gobionelline-like, Mugilogobius group	–	Gobiinae, Ptereleotridae
*Ponticola kessleri*	gobiine-like, Benthophilinae	–	–	–	–	–	Benthophilinae, Ponticolini
*Pseudogobius javanicus*	gobionelline-like, Mugilogobius lineage	–	–	–	–	gobionelline-like, Mugilogobius lineage	–
*Rhyacichthys aspro*	outgroup, Rhyacichthydae	–	–	outgroup	–	Rhyacichthyidae	outgroup
*Scartelaos histophorus*	gobionelline-like, Periophthalmus lineage	gobionelline-like, Periophthalmus group	gobionelline-like	–	gobionelline-like, Periophthalmus group	gobionelline-like, Periophthalmus lineage	Oxudercinae, Amblyopinae
*Sicyopterus lagocephalus*	gobionelline-like, Stenogobius lineage	–	–	sicydiines-/(gobionelline-) like, Amphidromous gobies	–	gobionelline-like, Stenogobius lineage (sicydiines)	Gobionellinae, Sicydiinae
*Tridentiger bifasciatus*	gobionelline-like, Acanthogobius lineage	–	–	gobionelline-like, Northern Pacific Group	–	gobionelline-like, Acanthogobius lineage	–

Only species for which information on their classification was available from previous phylogenetic studies are included

Gobionelline-like gobiids split into two major groups, with members from the *Acanthogobius*- and *Mugilogobius*-lineages (*sensu* Agorreta et al. [15]) in one, and members from the *Stenogobius-* and *Periophthalmus-*lineages (*sensu* Agorreta et al. [15]) in the other group. The sand goby clustered as sister taxon to all other gobionelline-like gobiids. Since statistical support was too low to determine the exact branching order among all three major groups within gobionelline-like gobiids, the sand goby may cluster as sister taxon to either one of the two other large gobionelline-like gobiid groups.

Gobiine-like gobiids clustered into two major groups. Unexpectedly, one of those contained *Micropercops swinhonis*, a member of Odontobutidae [26], and *Oxyurichthys formosanus*, which was expected to cluster with the gobionelline-like lineage *Stenogobius* [15], with high statistical support. The round goby clustered with the bighead goby, the only other West Eurasian gobiine-like gobiid species included into this study. These two species grouped with two *Glossogobius* species, albeit with low statistical support.

### Linking ecology, biology, and mitochondrial phylogeny

To complement the molecular phylogeny, we mapped genetic, biological, and ecological traits such as mitochondrial genome size, gene rearrangements, geographical occurrence, body length, preferred salinity, and specialized adaptations to the mitochondrial phylogenetic tree. We found that mitochondrial genome size, body length, and specialized adaptations were linked to particular branches with high statistical branch support, while the other features were independent from the mitochondrial phylogeny.

Extremely large mitochondrial genomes were distributed randomly throughout the gobiid mitochondrial phylogeny, but smaller-than-average and larger-than-average genomes clustered together with high statistical branch support. The four species with exceptionally large mitochondrial genomes exceeding 17 kb, round goby, *Chaeturichthys stigmatias*, *Amblychaeturichthys hexanema*, and *Odontobutis platycephala*, clustered with the gobiine-like gobiids, the gobionelline-like gobiids, and Gobiidae outgroups, respectively. However, when considering only mitochondrial genomes smaller than 17 kb, smaller genomes (up to 16,600 bp) were particularly prevalent in an *Acanthogobius*/*Mugilogobius-*lineage (e.g. *Gillichthys mirabilis*, *Pseudogobius javanicus*) while larger genomes were more common in a clade formed exclusively by species related to members of the *Acanthogobius-*lineage (e.g. *Acanthogobius hasta*) and in a clade consisting of members of the *Periophthalmus*-lineage (e.g. *Odontamblyopus rubicundus*) (lineage designations *sensu* Agorreta et al. [15]; Fig. 4).

For the ecological parameters compiled, we found that salinity preference and geographical occurrence were independent from the mitochondrial phylogeny, while body size and specialized adaptations occurred within certain clades or groups for the species included in this study that received high statistical branch support. Specifically, many of the analyzed members of the *Rhinogobius* group (gobionelline-like gobiids) were small. Burrowing life-style appeared to be a feature of gobionelline-like gobiid species only. Nonetheless, the trait appeared to have evolved twice independently based on the mitochondrial phylogeny. Similarly, air-breathing and amphibious life style was restricted to the gobionelline-like clade, but appeared to have several independent origins.

## Discussion

### Functional implications of the round goby sequence insertions

While sequence variations and repetitive elements flanking the control regions are common in a wide range of species [27–29], the non-coding sequence inserted downstream of tRNA Phenylalanine in the round goby mitochondrial genome is located at an unusual position and may therefore either come at a cost and/or have a novel function. Repeated sequence motifs and a degenerate copy of tRNA Phenylalanine at the end of the insertion suggest that the insertion arose from a duplication event involving the 3’ end of the control region. Since the insert does not contain TAS or CSB-like sites, it is quite unlikely that the insert represents a functional duplicated control region as observed in parrots [21], mites and ticks [30], silk moths [31], or millipedes [32]. However, the insert may be transcribed into RNA, since the origins of heavy strand transcription lie upstream of tRNA Phenylalanine [33–35].

Transcription and replication are rate limiting steps in tissues with high energy demands [35]. Therefore, animal mitochondrial genomes are under selection for small size and are depleted of non-coding sequences, they lack introns and intergenic sequences (discussed in [20, 21]). The retention of a non-coding sequence in the round goby thus suggests functional relevance. Mitochondrial variants can have great impact on the fitness of an organism (reviewed in [19, 35]). Also, size selection on mitochondrial genomes is stronger in endotherms than in ectotherms, which indicates that metabolic rates and mitochondrial genotypes may be closely linked [20]. In this context, it is of particular interest that the highly invasive round goby has lower metabolic rates, and controls metabolic rates better at high temperatures than other less invasive Ponto-Caspian goby species [36].

### Phylogenetic origins of mitochondrial genome size

Our results indicate that the tendency to harbour a smaller- or larger-than-average mitochondrial genome may be a feature of entire mitochondrial lineages. Mitochondrial genome size has been linked to body temperature and metabolism [20]. Thus, the observed size patterns may be linked to ecological parameters such as water temperature that were not covered in this study. Alternatively, the propensity of certain lineages to generate and tolerate sequence insertions on the one hand or to select for small mitochondrial genome size on the other hand may possibly depend on evolutionary differences in the DNA replication or repair machineries of those lineages.

### Potentials and limitations of gobiid mitochondrial genome phylogenies

Our phylogenetic reconstructions recovered the two previously described clades within Gobiidae, gobiine-like gobiids and gobionelline-like gobiids [15], and confirmed the placing of round goby in the former and sand goby in the latter group [3, 15–17, 37]. For gobionelline-like gobiids, our results agree with previous studies, except for members of the *Mugilogobius*-lineage. We find *Mugilogobius* species nested within an *Acanthogobius*-lineage, while they were considered sister groups, albeit with low statistical support, by two recent studies [3, 15]. The gobiine-like gobiid clade also contains two unexpected members: *Oxyurichthys formosanus* and *Micropercops swinhonis*. Previously, other members of the *Oxyurichthys* genus grouped with gobionelline-like gobiids (e.g. *Oxyurichthys stigmalophius*; [15], *Oxyurichthys lonchotus* and *Oxyurichthys ophthalmonema* [3]). *Micropercops swinhonis* was previously placed with Odontobutidae [26], which form a sister lineage to Gobiidae [38]. However, *Micropercops swinhonis* does not always group with *Odontobutis* [39], and its developmental process is different from *Odontobutis* but resembles that of Gobiidae [39]. Also, it contains the same His-Ser-Leu tRNA arrangement as all Gobiidae, while *Odontobutis platycephala* shows a unique Ser-Leu-His arrangement. Thus, *Micropercops swinhonis* may be a true member of Gobiidae.

For sand goby, our results reflect previous uncertainties about the exact phylogenetic placement of this species. The position as sister taxon to either an *Acanthogobius/Mugilogobius* lineage, to a *Stenogobius/Periophthalmus* lineage, or to all four gobionelline-like gobiid lineages is consistent with [37] (sister taxon to *Acanthogobius* species), [3] (sister taxon to *Mugilogobius/Acanthogobius*), [17] (sister taxon to *Mugilogobius*)*,* but conflicting with [40] (sister taxon to all gobiine-like gobiids), and [16] (clustering with other members of the *Pomatoschistus-*lineage among gobiine-like gobiids).

As expected, the round goby groups together with the *Ponticola kessleri*, the bighead goby, which is at present the only other representative of Benthophilinae with an available complete mitochondrial genome. The Ponto-Caspian species group and their immediate relatives have been previously suggested to have experienced an evolutionary burst that led to the formation of three Benthophilinae tribes, Ponticolini, Neogobiini, and Benthophilini [16]. Both round goby (Neogobiini) and bighead goby (Ponticolini) feature a particular tRNA arrangement (Gln-Ile-Met instead of the common Ile-Gln-Met arrangement). If this arrangement is also present in Benthophilini, it may be a specific signature of the entire group, and may be related to the suggested historic radiative event promoting the diversification of Benthophilinae. The differential orientation of these tRNAs on the heavy and light strand in the round goby and the bighead goby may in turn help to shed light on the radiation within Benthophilinae.

Many previous phylogenetic studies of gobiids were based on a combination of individual nuclear and mitochondrial markers or on nuclear markers alone. Differences among topologies obtained from different regions of the genome (“gene trees”) are expected and can be explained by different evolutionary processes acting on those regions, such as incomplete lineage sorting, gene duplications and hybridization [41–43]. All in all, our results strongly speak for further examination of Gobiidae, in particular West Eurasian gobiids, using denser taxon sampling for the mitochondrial genome and additional nuclear markers.

### Ion-transport capacities may explain the tremendous success of Gobiidae

One striking feature of gobiids is their capacity to colonize very different habitats and even the land. We support the idea that this capacity, albeit it manifests itself in restricted lineages only, may be linked to a more ancient ability of this family to deal with fluctuating ion concentrations. Euryhalinity and amphidromous life cycle are present in many gobiid clades, and species with a preference of high or low salt conditions are evenly distributed across the mitochondrial phylogeny including outgroup species from other Gobioidei lineages, indicating that adaptation to novel salinity levels may be a capacity of all gobioids. Similarly, several gobioid mitochondrial lineages contain amphidromous species, indicating that the ability to breed in fresh water, develop in sea water, and then return to freshwater may be an ancient adaptation in the gobioid group. In the gobionellinae-like gobiids, amphidromous species belong to the *Stenogobius*- and *Acanthogobius*-lineages (*sensu* Agorreta et al. [15]), represented by *Sycopterus*, *Stiphodon* and *Rhinogobius* in the present study, and in gobiine-like gobiids to *Glossogobius* [44]. The amphidromous life cycle is additionally present in the gobioid families Eleotridae and Ryacichthyidae, which are sister taxa to Gobiidae in the gobioid phylogeny.

In contrast, the ability to breathe air is mostly restricted to the *Periophthalmus*-lineage (*sensu* Agorreta et al. 2013; recovered with high statistical branch support in this study). *Scartelaos*, *Boleophthalmus* and *Periophthalmus,* the so-called mudskippers, spend a large time of their time above water [45]. In agreement with [15] and [46], mudskippers are a paraphyletic group in the mitochondrial phylogeny and appear to have evolved twice independently. Alternatively, the mud-burrowing ‘eel gobies’ represented by *Odontamblyopus rubicundus* and *Trypauchen vagina* in the present study, may have evolved from mudskippers. Also, the mud flat living non-mudskipper *Oxyderces dentatus* may actually have a partly amphibious lifestyle [45]. Thus, all members of this lineage follow land- and air-oriented lifestyles.

While euryhalinity and air breathing may seem like very different features, euryhalinity, amphidromous life cycle, and amphibious life style all expose organisms to salinity oscillations, and require the ability to tolerate and excrete ammonia independently of ambient levels. Thus, they all demand excellent and adaptable active ion transport capacities [45, 47]. These observations suggest that the entire gobiid family may have evolved the ability to deal with fluctuating ion gradients, which in turn may be the key to their world-wide success.

## Conclusion

In conclusion, we find that the sand goby mitochondrial genome closely resembles other gobiid mitochondrial genomes. The round goby on the other hand has an unusually large mitochondrial genome featuring tandem repeat expansions in the control region and a non-coding and potentially transcribed sequence inserted downstream of the putative origin of transcription. We may thus speculate that the tremendous colonization abilities of this species may be linked to special features of its mitochondrial metabolism. While we were not able to further resolve the phylogenetic placement of the sand goby and the round goby, we provided additional information on the placement of *Oxyurichthys formosanus* and *Micropercops swinhonis* in the mitochondrial phylogeny*.* Also, we identified a novel molecular marker for Ponto-Caspian species in the gene arrangement of the tRNAs Gln-Ile-Met. From analysing the ecological traits of air-breathing and amphibious lifestyle we speculate that the entire species group may be adapted to deal with challenging ion transport conditions, which may be the critical factor in their world-wide success.

## Methods

### Sequencing of the round goby mitochondrial genome

The round goby mitochondrial genome was obtained by PacBio single molecule sequencing. Genomic DNA was extracted from the liver of one male individual of round goby caught in Basel, Switzerland (47° 35′ 18″ N, 7° 35′ 26″ E) in spring 2015. At the Genome Center Dresden, Dresden, Germany, 300 mg of liver tissue were ground by mortar and pestle in liquid nitrogen and lysed in Qiagen G2 lysis buffer with Proteinase K. RNA was digested by RNase A treatment. Proteins and fat were removed with two cycles of phenol-chloroform extraction and two cycles of chloroform extraction. Then, the DNA was precipitated in 100% ice cold Ethanol, spooled onto a glass hook, and eluted in 1x TE buffer and stored at 4 °C. 10 μg of DNA were cleaned up on AMPure beads. From this DNA, five long insert libraries were prepared for PacBio sequencing according to the manufacturer’s protocols. Genomic DNA was sheared to 35 kb using the Megaruptor device. The PacBio libraries were size selected for fragments larger than 15 kb using the BluePippin device. PacBio SMRT sequencing was performed with the P6/C4 chemistry using 240 min sequencing runs.

### Sequencing of the sand goby mitochondrial genome

The sand goby mitochondrial genome was obtained by Illumina sequencing. Genomic DNA was extracted from the tail fin of one individual male *Pomatoschistus minutus* caught in summer 2010 in Bökevik Bay (58° 14′ 55″ N, 11° 26′ 51″ E) close to the Sven Lovén Centre For Marine Sciences, Kristineberg, Sweden. The tissue was digested in CTAB-buffer, mercaptoethanol and Proteinase K [48]. RNA was digested by RNase A treatment. Thereafter, DNA was purified by chloroform-isoamylate extraction and precipitated with isopropanol. The DNA was washed with 70% ethanol and stored in 10% TE-buffer at -70 °C. One 300 bp paired-end library was sequenced on an Illumina HiSeq2000 (2x101 bp) at the national sequencing facility, Science for Life Laboratory, in Stockholm, Sweden.

### Assembly of the round goby mitochondrial genome

The round goby mitochondrial genome was assembled at the Heidelberg Institute for Theoretical Studies HITS gGmbH. First, consensus reads covering mitochondrial sequences were pulled out via the Pacbio long read aligner (blasr [49]) using the bighead goby mitochondrial genome [50] as query. Local multiple alignments of the mitochondrial reads were computed with daligner [51]. From the resulting overlaps, five high quality reads were selected as template genomes for the error correction step. Then, these were extended on both sites by the overhang sequence of overlapping highest quality reads to ensure that each read contained at least one copy of the mitochondrial genome. Errors were corrected with PacBio’s quiver program [52]. Thereafter, all mitochondrial reads were mapped back onto the five mitochondrial template genomes with blasr. Coverage was on average 170x. The resulting consensus sequences were aligned in CLC Main Workbench 6.8.1 (https://www.qiagenbioinformatics.com/). The resulting consensus was further corrected by majority vote. Open reading frame shifts after homopolymers were attributed to sequencing errors and were manually corrected by adapting homopolymer length.

### Assembly of the sand goby mitochondrial genome

The sand goby mitochondrial genome was assembled at 1573x coverage in CLC Main Workbench 4.06beta.67189 (https://www.qiagenbioinformatics.com/). A second assembly was performed using SOAPdenovo v1.3 (http://soap.genomics.org.cn/).

### Verification of the round goby mitochondrial genome

We verified the round goby mitochondrial genome by two methods. First, we aligned the round goby and the bighead goby mitochondrial genomes in ClustalOmega [53] and assumed that conserved regions were correctly sequenced. Second, we identified non-conserved regions and confirmed them by PCR and Sanger sequencing on five additional round goby individuals caught in Basel, Switzerland, in 2015. The first such region spanned the tRNAs Glutamine, Isoleucine, and Methionine. The second such region spanned the non-coding region between tRNA Proline and the 12S gene. Overlapping amplicons were designed to amplify and sequence those regions. Primer sequences were, 5′–3′: fw1, CCCGATTCCGATATGACCAAC; rev1, GGCTGGATTTTAACCGGCATG; fw2, AGAGCGCCGGCCTTGTAAG; rev2, CAGGTCTTAACTTGGTGTGAG; fw3, ACCCAACTCGAGATTTTCCTG; rev3, CATCAACAATCATTCAAGAATGC; fw4, ATATCATGAGCATAAGTAATTGAC; rev4, GATTGGGTGCAGATCACAGTG; fw5, TACAAAATTGCCCATAATTATGAC; rev5, GGGGTGAGGAGACTTGCATG. We purified DNA from lateral muscle with the Qiagen Blood and Tissue kit. PCR was performed on 50 ng of DNA using FastStart Taq DNA Polymerase from Roche. PCR conditions for amplicon 1 (fw1 and rev1) were: 4′ at 94 °C; 35 cycles of 30″ denaturing at 94 °C, 30″ annealing at 56 °C, 40″ or 1 min/kb extension at 72 °C; 5′ at 72 °C. PCR conditions for amplicons 2, 3, 4, and 5 were adapted for amplification of AT-rich sequences [54] so that extension temperature was 65 °C for amplicons 2, 4, and 5, and 72 °C for amplicon 3, and so that extension time was 2 min for all four amplicons. Amplified fragments were TA-cloned and sequenced at Microsynth, Switzerland. All resulting sequences were identical to those from the PacBio based assembly.

### Verification of the sand goby mitochondrial genome

Since repetitive regions which are often present around the mitochondrial control region are difficult to assemble from short reads, the sand goby mitochondrial genome was assembled a second time from 300 bp paired-end reads from a 500 bp insert library, with identical results. We did not identify regions of higher than average coverage in the control region, which would indicate collapsed repeats.

### Comparing mitochondrial genome lengths

To understand whether the size of the round goby and sand goby mitochondrial genomes was in the normal range for fish and vertebrates, we compared them to other available mitochondrial genomes. We collected complete mitochondrial genomes of animals, vertebrates, and bony fish from the NCBI database, using the search terms “vertebrate mitochondrion complete genome”, “animalia mitochondrion complete genome”, and “actinopterygii mitochondrion complete genome”. Partial mitochondrial sequences were removed from the dataset in R [55]. Some species, such as human or mouse, were overrepresented in the dataset. Also, for some species with more than one mitochondrial genome available, entries differed in length. To avoid overrepresentation bias and to obtain a single length value for each species, a median length was calculated for each species in R [55]. Those median values were plotted by taxonomic class as obtained from the IUCN database [56].

### Gene arrangement analysis

To analyse gene arrangement conservation in an unbiased manner, we annotated or re-annotated all complete mitochondrial goby genomes available on NCBI using MitoFish v2.95 [57]. Species names and accession numbers are listed in Additional file 1: Table S1. Annotations were then visually screened for deviations from the common gene arrangement.

### Annotation of round and sand goby mitochondrial genomes

We annotated the sand and round goby mitochondrial genomes using MitoFish v2.95 [57]. We corrected the automated annotation manually in Geneious 5.6.7 [58] using an alignment with complete mitochondrial genomes from *Ponticola kessleri*, *Amoya chusanensis*, *Glossogobius circumspectus*, *Glossogobius olivaceus*, *Oxyeleotris marmorata*, *Siganus guttatus*, *Mogurnda adspersa*, *Bostrychus sinensis*, *Scartelaos histophorus*, *Boleophthalmus boddarti*, *Odontamblyopus rubicundus*, *Oxuderces dentatus*, and *Lactoria diaphana*, including their annotations (see Additional file 1: Table S1 for accession numbers).

### Analysis of non-coding sequence arrangements

To understand the evolution of the novel non-coding sequences in the round goby mitochondrial genome, we compared the non-coding regions of sand goby, round goby, and its closest sequenced relative, the bighead goby. Repetitive regions were identified with the dot plot tool of CLC Main Workbench 6.8.1 with window lengths of 10 nucleotides (nt) and 15 nt. Sequence repeats were identified through manual searches using consecutive 10 nt-blocks of the non-coding sequences as query and all three non-coding regions as target. The identified repeats were extracted and aligned in CLC biobench to generate a consensus for each repeat. Since mitochondrial non-coding sequence repeats have been shown to form secondary structures with functions in protein binding, replication or transcription [59–61], we tested the ability of the consensus sequences to form secondary structures with a minimum free energy approach based on the Zuker algorithm [62] implemented in CLC Main Workbench 6.8.1.

To identify functional regions in the non-coding sequence blocks, we searched for the conserved sequence blocks, CSB I to III, and the termination associated site, TAS [27, 63–65]. To this end, we extracted the sequences between tRNA Proline and tRNA Phenylalanine from the analyzed species, aligned them with ClustalOmega [53] and identified regions of high conservation with the Percentage Identity coloration of Jalview 2.9.0b2. Then, we searched for TAS motifs using the query ATGN(8–9)CAT [65]. We searched for CSB-like motifs [27] using the following queries: CSB I, AT or ATG followed in some distance by GACA; CSB II, AAACCCCCCNNNCCC; CSB III, AAACCCC within an otherwise A/C-homopolymer rich region.

### Phylogenetic analyses

We aligned the annotated mitochondrial genomes of forty Gobiidae species, nine species from other gobioid families, and of *Siganus guttatus* and *Lactoria diphana* with the round and the sand goby mitochondrial genomes using Geneious 5.6.7 [58]. We kept only protein-coding regions in the alignment, excluded the gene ND6, which is evolving under different constraints than other mitochondrial genes [66], and manually removed all gaps, double checking for correct reading frames using the translated amino acid sequences. We then used MEGA7.0.14 [67] to determine the best-fitting model of sequence evolution. Thereafter, two independent phylogenetic trees were calculated, one based on Bayesian inferences and one based on maximum likelihood analyses. For Bayesian inferences in MrBayes 3.2 [68], we used one cold and three heated chains and ran the analyses for 10,000,000 Markov chain Monte Carlo generations sampling every 2′000th generation, with a burnin of 25%. Convergence was confirmed in Tracer v1.5 (effective sampling size > 200). For maximum likelihood analyses in raxmlGUI 1.3.1 [69], we used the rapid hill-climbing algorithm [70] with 1000 bootstrap replicates [71].

### Mapping ecological properties to the phylogenetic tree

We retrieved information on body length, preferred salinity, geographical distribution, and specialized adaptations for all analysed gobioid species from FishBase [72]. We then verified the information using published scientific literature (see Additional file 1: Table S1 for references) and mapped it to the phylogenetic tree.

## Additional files

Additional file 1: Table S1. Contains information on the sequences used for phylogenetic analysis and the references used for each species to extract biological and ecological characteristics. **Table S2.** Contains median mitochondrial genome sizes for ray finned fish. **Table S3.** Contains median mitochondrial genome sizes and class annotations for vertebrates. (XLSX 126 kb)
Additional file 2: Figure S1. This figure shows the aligned non-coding sequences of all gobioids included into this study, with the query sequences of TAS and CSB elements indicated above the alignments, and hits for this query coloured in the alignment. (PDF 25604 kb)
Additional file 3: Figure S3. This figure shows graphical representations of the MitoFish annotation results for all gobioid mitochondrial genomes. (PDF 28930 kb)
Additional file 4: Figure S2. This figure shows sequence alignments of the individual occurrences of the repeat motifs identified in the round and the bighead goby, the extracted consensus sequence and the predicted secondary structure of the consensus. (PDF 1954 kb)

## Abbreviations

CSB: Conserved Sequence Block
TAS: Transcription Associated Site
