# Supplementary figures and images for: The mitochondrial genome sequences of the round goby and the sand goby reveal patterns of recent evolution in gobiid fish
Source: BMC Genomics. 2017 Feb 16;18:177. doi: 10.1186/s12864-017-3550-8 (PMC5314710; doi:10.1186/s12864-017-3550-8)

TAS

ATG N(8) CAT

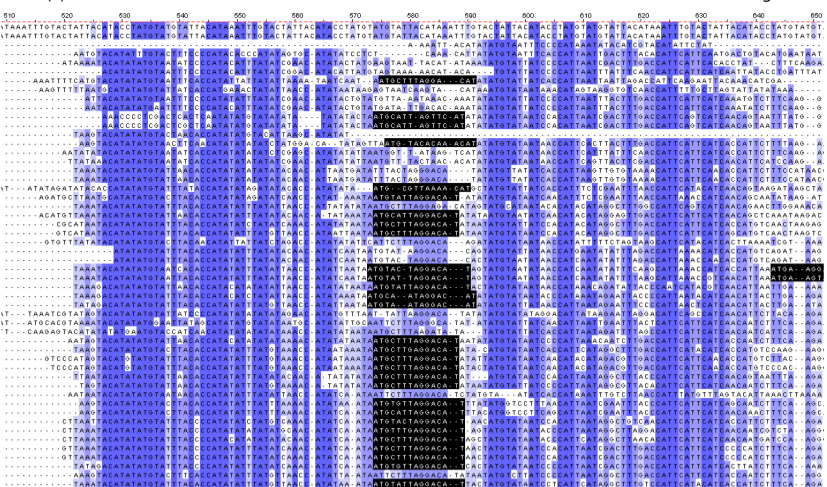

ATG N(9) CAT

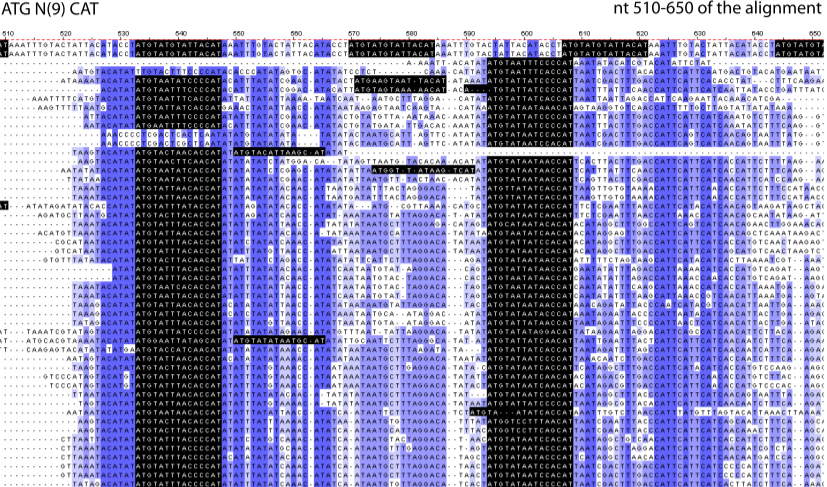

CSB I

ATG N(8-12) GACA

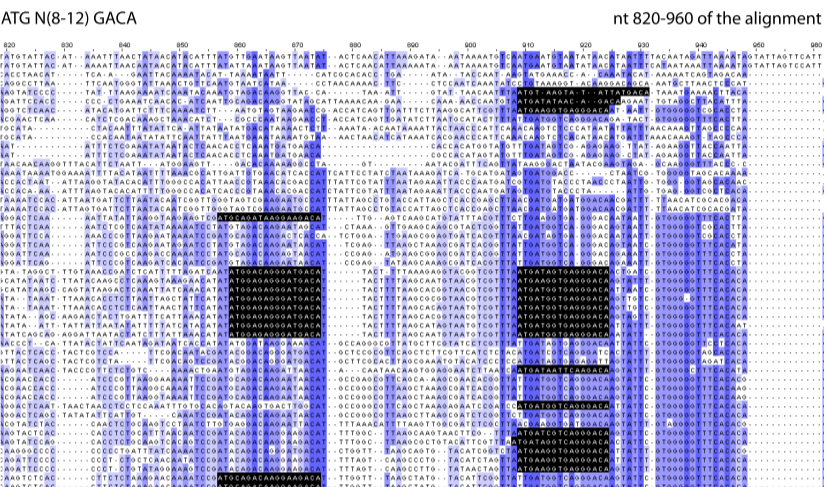

AT N(8-13) GACA

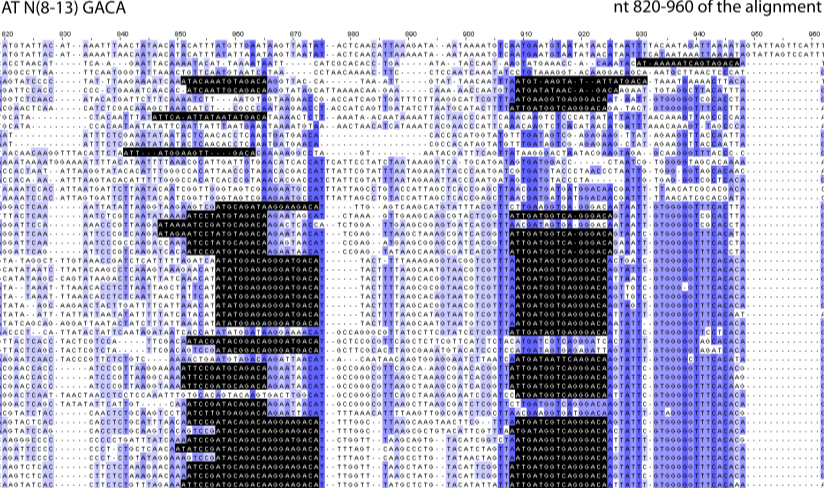

CSB II

AAACCCCN(3)CCC

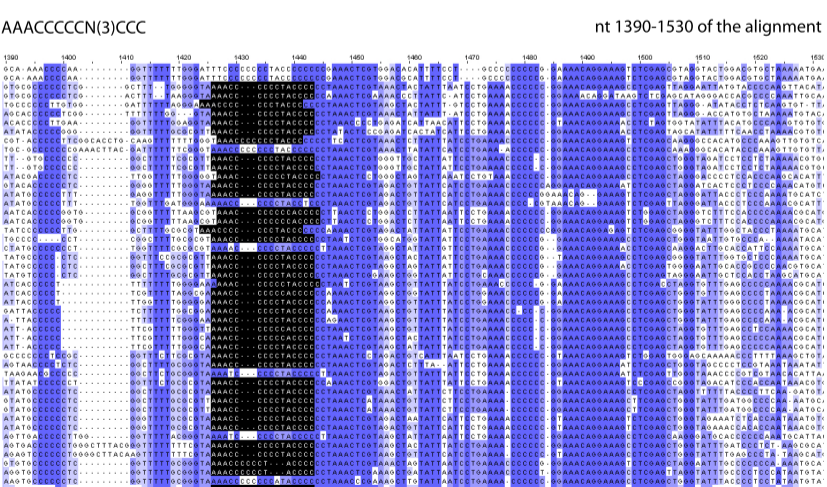

CSB III

AAACCCC

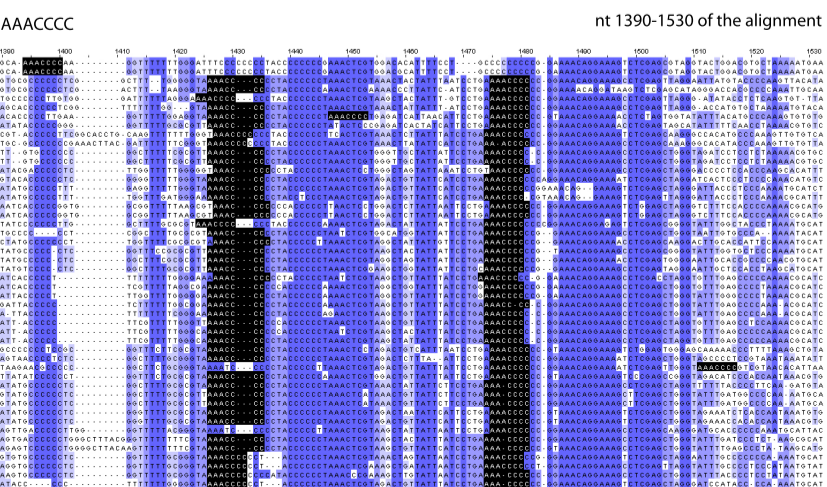

Supplement: Additional file 2: Figure S1. — This figure shows the aligned non-coding sequences of all gobioids included into this study, with the query sequences of TAS and CSB elements indicated above the alignments, and hits for this query coloured in the alignment. (PDF 25604 kb) [file 12864_2017_3550_MOESM2_ESM.pdf]

Supplementary Figure 3

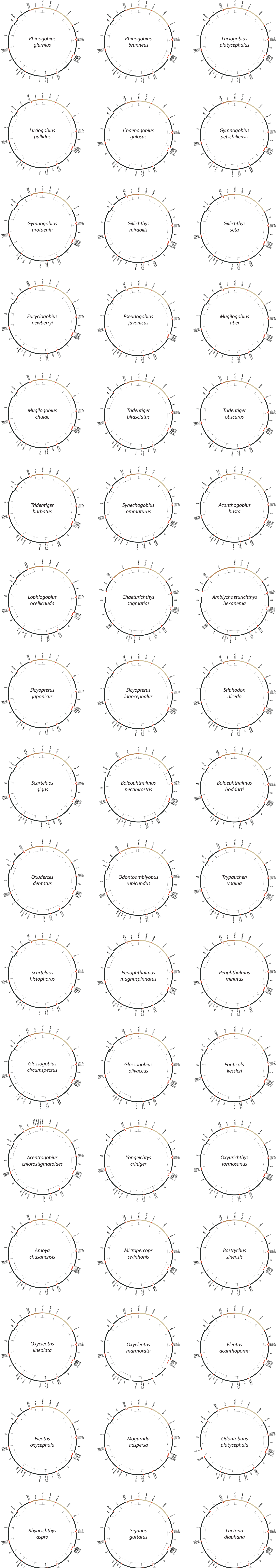

Supplement: Additional file 3: Figure S3. — This figure shows graphical representations of the MitoFish annotation results for all gobioid mitochondrial genomes. (PDF 28930 kb) [file 12864_2017_3550_MOESM3_ESM.pdf]

Supplementary Figure 2

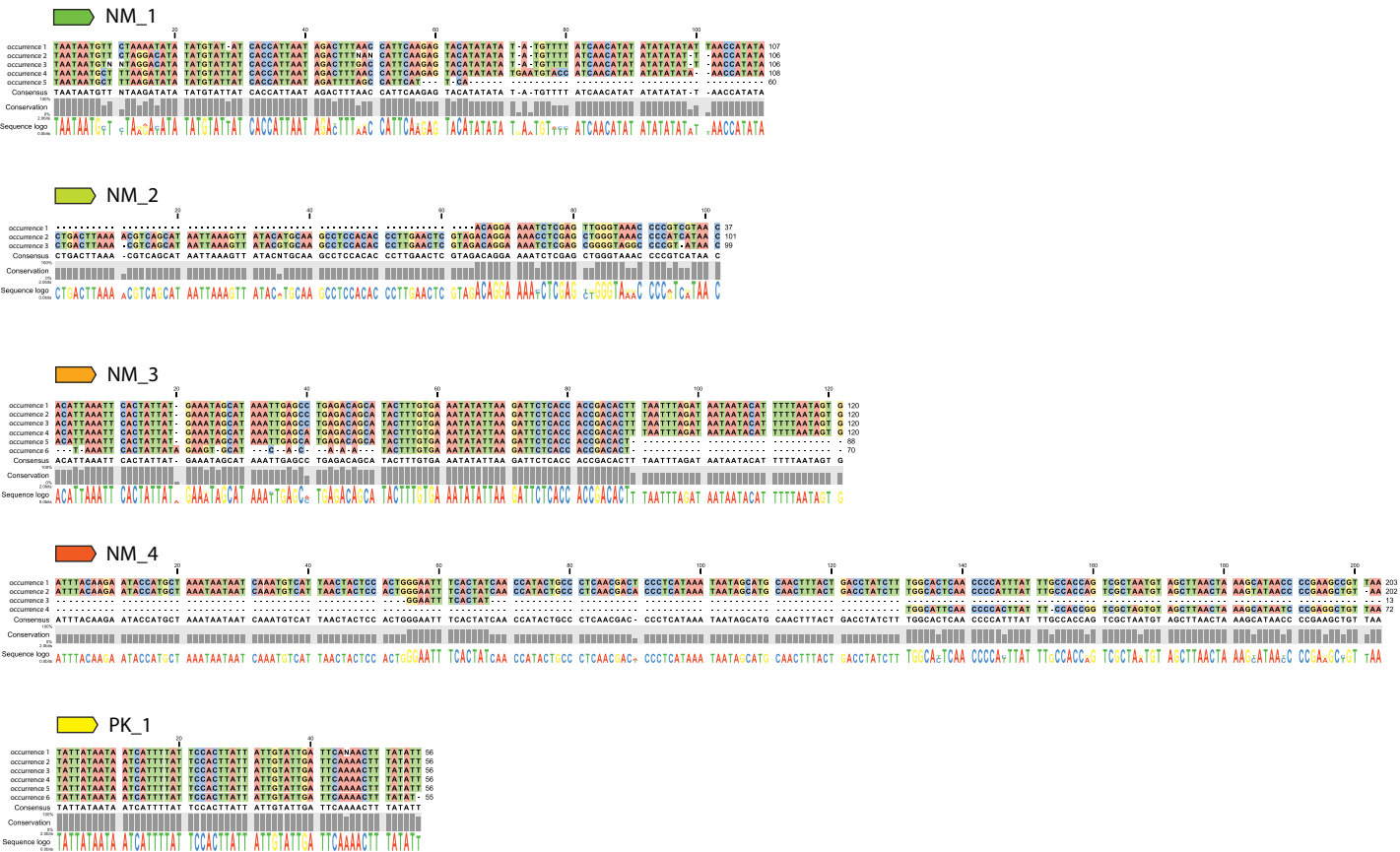

round goby

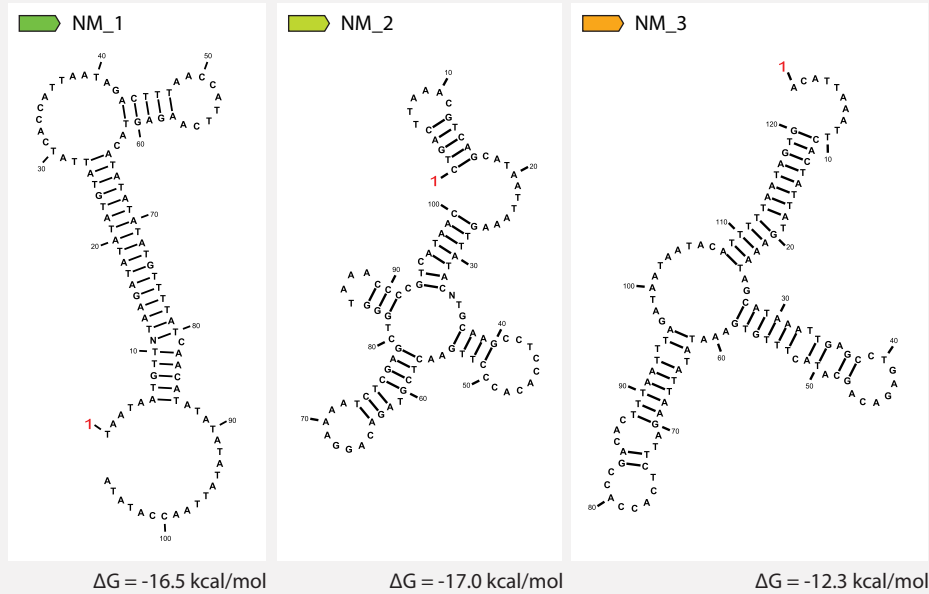

bighead goby

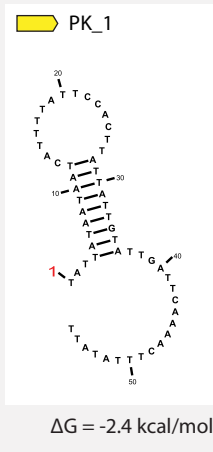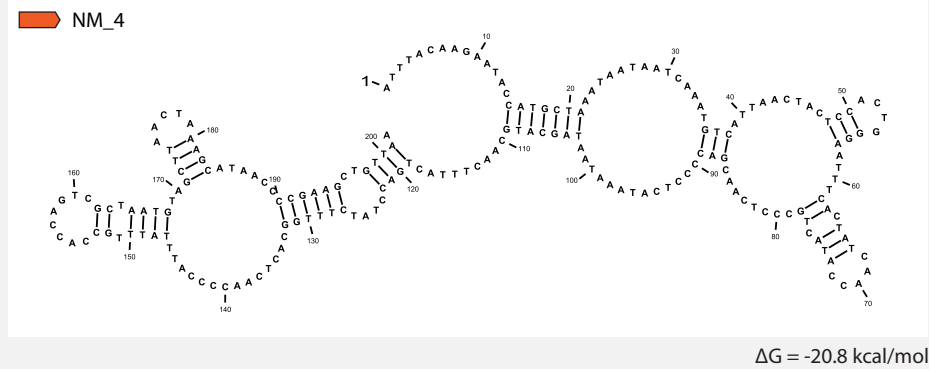

Supplement: Additional file 4: Figure S2. — This figure shows sequence alignments of the individual occurrences of the repeat motifs identified in the round and the bighead goby, the extracted consensus sequence and the predicted secondary structure of the consensus. (PDF 1954 kb) [file 12864_2017_3550_MOESM4_ESM.pdf]
